# Cardiovascular Manifestations in Inflammatory Bowel Disease

**DOI:** 10.2174/011573403X256094231031074753

**Published:** 2023-11-24

**Authors:** Anish Kumar Reddy Meda, Fremita Chelsea Fredrick, Urvashi Rathod, Priyanshi Shah, Rohit Jain

**Affiliations:** 1 Avalon University School of Medicine, Willemstad, Curacao;; 2 Narendra Modi Medical College, Ahmedabad, India

**Keywords:** Crohn's disease, ulcerative colitis, cardiovascular disease, inflammatory bowel disease, endotoxemia, pericarditis, thromboembolism

## Abstract

Inflammatory bowel disease is a group of long-term systemic inflammatory disorders affecting the gastrointestinal tract, including Crohn's disease and ulcerative colitis, which may be associated with an increased risk of developing extraintestinal manifestations, including cardiovascular disease, thereby decreasing the quality of life. Pathophysiological changes associated with inflammatory bowel disease include alterations of the microbiome, endotoxemia, and changes to glucose and lipid metabolism. Inflammatory bowel disease patients have higher carotid intima-media thickness, lower flow-mediated dilatation, and increased carotid-femoral pulse wave velocity, which are markers of elevated cardiovascular risk. In addition, inflammatory bowel disease patients are at an increased risk for developing venous and arterial thrombotic events due to a hypercoagulable state caused by thrombocytosis and coagulation system activation. To reduce the risk of developing cardiovascular disease, lifestyle modifications, such as smoking cessation, dietary changes, and increased physical activity alongside management with appropriate medication, should be considered. This paper examines how inflammatory bowel disease can influence the risk of cardiovascular complications and the involvement of drug therapy.

**Methods:**

PubMed was searched using keywords, such as inflammatory bowel disease, Crohn's disease, ulcerative colitis, cardiovascular disease, pericarditis, thromboembolism, and many more. Relevant literature up to March 2023 has been examined and summarized, which consisted of data from various clinical trials, meta-analyses, retrospective/prospective cohort studies, and current guidelines.

## 1 INTRODUCTION

Inflammatory bowel diseases (IBD) are chronic systemic inflammatory disorders that primarily affect the gastrointestinal tract and are clinically categorized as Crohn's disease (CD), ulcerative colitis (UC), or unclassified IBD [1]. Over recent years, a marked increase in the prevalence of these conditions has been observed globally, with UC being the most common form of IBD and generally involving the rectum, part of the colon proximal to the splenic flexure, or the entire colon from the proximal to the splenic flexure, while CD inflammation has a propensity to be found within the small intestines (particularly the ileum), large intestines, and perianal area [2-6]. The prevalence of IBD in North America is currently 0.3% and is projected to reach 0.6% by the end of the decade, equating to an estimated 2.2 million IBD patients, with a female predominance in Western populations and male predominance in eastern populations [7]. African American, Hispanic, and Asian patients have been reported to have a higher prevalence of IBD when compared to Caucasians [8]. In addition, adults with less than a high school level of education, unemployment, poverty, or suburban locality are also known to have an increased predisposition to IBD [9]. The utilization of immunomodulators, anti-tumor necrosis factor alpha agents, anti-interleukin agents, interferons, immune-boosting agents, and checkpoint inhibitors is correlated with developing secondary IBD. Furthermore, procedures, such as a colectomy, may precipitate *de novo* CD, ileal pouchitis, or post-colectomy enteritis syndrome, and patients may experience de novo IBD or an IBD flare following organ transplant or hematopoietic stem cell transplantation. Fecal microbiota transplantation is another standard procedure for treating recurrent Clostridium difficile infections and may provoke IBD exacerbation [10]. Extraintestinal manifestations (EIMs) of IBD are experienced by 5-50% of patients, with 29% of cumulative EIM symptoms arising after 15 years of the disease development [11, 12]. Additionally, an elevated risk of cardiovascular morbidity has been associated with IBD, with an 18% higher risk for cerebrovascular accidents (particularly in females and young patients) and an 18% higher risk for ischemic heart disease (particularly in females) when compared to individuals without IBD [13]. Cardiovascular disease (CVD) is a leading cause of morbidity and mortality, with coronary heart disease and stroke being the most common, and is associated with risk factors, such as obesity, hypertension, and diabetes [14]. The relationship between IBD and atherosclerotic cardiovascular diseases (ASCVD), an inflammatory condition of the arteries linked with cholesterol and other metabolic changes, is still not clear-cut, yet studies have suggested that chronic inflammatory conditions, such as rheumatoid arthritis, systemic lupus erythematosus, and ankylosing spondylitis, may contribute to an increased risk of CVD [15, 16]. Chronic inflammatory diseases are a major contributor to morbidity and a known cause of poor quality of life and shorter life expectancy globally; they are identified as non-infectious illnesses in which chronic inflammation has a crucial role in the etiology and progression of the diseases. It is speculated that up to 90% of various chronic diseases could be prevented with improvements to lifestyle factors, such as smoking, dietary choices, and physical inactivity [17]. In this research paper, we have explored how IBD can influence the risk of cardiovascular complications and considered the possible treatment options.

## 2 PATHOPHYSIOLOGY

**Fig. (1):**
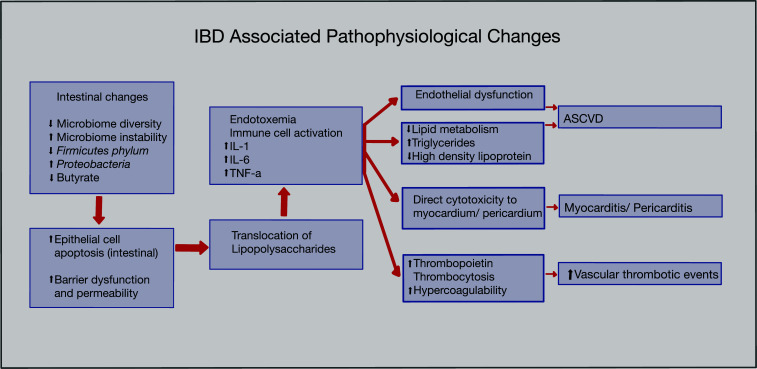
Schematic representation of the pathophysiological changes in inflammatory bowel disease leading to cardiovascular manifestations.

IBD has a variety of pathophysiological changes that can lead to an increased risk of developing CVD. An alteration of the intestinal microbiome can lead to a less diverse and unstable microbiota, in which the *Firmicutes* species and butyrate production are decreased, whereas the *Proteobacteria* species are increased compared to healthy controls [18]. Butyrate production is mainly necessary to assemble tight junctions and enhance the intestinal barrier [19]. Hence, decreased butyrate production leads to increased intestinal barrier permeability and translocation of lipopolysaccharide into the circulation, which results in endotoxemia and the subsequent release of inflammatory cytokines, like interleukin (IL)-1, IL-6, and tumor necrosis factor-alpha (TNF-α) (Fig. **1**) [20]. Enhanced levels of these cytokines, C-reactive protein (CRP), and homocysteine in the context of IBD can also contribute to endothelial dysfunction [21]. Endotoxemia also causes alterations in glucose and lipid metabolism, causing increased triglycerides, increased insulin resistance, and decreased high-density lipoproteins, thereby increasing the risk of developing ASCVD, obesity, and diabetes [22]. In addition, since normal gut microbial enzymes (such as bile salt hydrolase and bile acid-inducible enzymes) are needed for deconjugation and dehydroxylation reactions in order for bile acid synthesis to occur, the altered microbiota may result in decreased production of these necessary enzymes, and thus cause a decreased synthesis of bile acids [23]. This may lead to an increased risk of developing dyslipidemia due to an accumulation of cholesterol and triglycerides [24]. IBD also results in direct cytotoxicity to the myocardium/pericardium, which causes exposure of usually hidden antigens to the immune system, leading to the activation of cross-reactive or autoreactive T-cells and induction of autoantibodies and eventual development of chronic myocarditis/pericarditis [25]. Chronic inflammation in the myocardium/pericardium can result in characteristic remodeling processes, such as dilation of the cardiac chambers (resulting in systolic dysfunction), rupture of papillary muscles (causing valvular regurgitation), and fibrosis of the conduction system (resulting in arrhythmias) [26]. A meta-analysis by Wu *et al.* demonstrated patients with IBD to have higher carotid intima-media thickness (CIMT), lower flow-mediated dilatation (FMD), and increased carotid-femoral pulse wave velocity (cfPWV) [27]. Each of these findings is a marker of elevated cardiovascular risk; CIMT is used as a surrogate marker for atherosclerosis, FMD assesses endothelial dysfunction, and cfPWV is used to measure arterial stiffness [28-30]. The exact pathophysiology of how these changes occur in IBD patients is unclear, but it is suggested that chronic inflammation and the immune response cause endothelial damage and structural changes in the arterial wall. It is important to note that the risk for atherogenesis and CVD is directly proportional to increased inflammation exposure time in IBD patients, further implying the connection between inflammation and Wu *et al*.’s findings. The hypercoagulable state of IBD results in a significantly higher risk of developing venous and arterial thrombotic events [31, 32]. This can be attributed to the development of thrombocytosis in IBD patients, which occurs as a result of increased bone marrow thrombopoiesis secondary to inflammatory mediators, such as IL-6, and increasing plasma concentrations of thrombopoietin [33]. The link between this IBD-related thrombocytosis and CVD can be confirmed by analyzing a study done by Thapa *et al.*, which compared 36 IBD patients with thrombocytosis to 72 controls, and found an increased risk of developing coronary artery disease in the IBD patients with thrombocytosis (36.1% *vs.* 9.7%, *P* = 0.02) [34]. Furthermore, inflammation in IBD causes changes in hemostatic biomarkers leading to activation of the coagulation system (due to tissue factor activation and increased procoagulant factors, like fibrinogen, von Willebrand factor, and factor V, VII, VIII, and XI), reduced activity of anticoagulant pathways (impaired protein C and S activation and decreased concentrations of antithrombin and tissue factor pathway inhibitor), and decreased activity of the fibrinolytic system (decreased tissue plasminogen activator-1 and increased fibrinolytic inhibitors, such as plasminogen activator inhibitor-1 and thrombin-activatable fibrinolysis inhibitor) [32]. Due to these various pathophysiological changes, diagnosis and treatment should be optimized in IBD patients at an early stage to prevent the development of cardiovascular complications.

## 3 DISCUSSION

IBD is frequently accompanied by symptoms, such as abdominal pain, fatigue, and diarrhea [35]. A variety of EIMs have been associated with IBD, including CVD (13.2%), peripheral arthritis (5-20%), eye inflammation (0.3-5%), and cutaneous manifestations (2-34%) [36-38]. The most common IBD-related cardiovascular complications include ASCVD, pericarditis, myocarditis, thromboembolism, ventricular impairment, arrhythmias, infective endocarditis, valvulopathy, and Takayasu arteritis [39, 40]. In a study conducted by Aniwan *et al*., it was discovered that individuals who have been diagnosed with IBD have a significantly higher cumulative incidence of heart failure (HF) when compared to individuals without IBD (*P* = 0.02) [41]. To better comprehend this correlation, it is crucial to take into account the relative risk (RR) linked to IBD and ischemic heart disease (IHD). For those with Crohn's disease (CD), the RR for IHD is 1.24, while for those with ulcerative colitis (UC), the RR is 1.21. Furthermore, females with IBD have a higher RR for IHD (1.35) compared to males (1.19) [42]. The heightened risk of IHD in patients with IBD leads to deleterious effects on the myocardium, including myocardial cell injury and death, ultimately predisposing these patients to the development of HF [41]. Additionally, studies have also suggested that exposure to systemic inflammation in individuals with IBD places them at an augmented risk of developing premature (<55 years) and highly premature (<40 years) ASCVD along with arrhythmias, such as atrial fibrillation [41, 43-45]. Although there are clear associations between CVD and IBD, the outcomes are interesting to note. A cross-sectional analysis conducted by Pemmasani *et al.* examined 24,200 hospitalizations for acute coronary syndrome in patients with IBD, which showed the mortality rate as lower in the IBD-acute coronary syndrome cohort (3.9%) compared to the non-IBD-acute coronary syndrome cohort (5.3%) [46]. On the contrary, another study revealed patients with both IBD and heart failure (HF) to have a higher risk of IBD-related hospitalizations [hazard ratio (HR) - 1.42), flares (HR-1.32), and complications (HR-1.32)] [47]. Additionally, during periods of persistent IBD activity and flares, there has been reported a 37% increased risk of hospitalization for HF. During periods of remission, however, the risk of hospitalization for HF has been found to be comparable to the reference population, suggesting that disease activity plays a role in this association [48]. In light of the contrasting findings from different studies, such as the lower mortality rate observed in hospitalized patients with IBD and acute coronary syndrome, and the increased risk of IBD-related hospitalizations, flares, and complications in patients with concurrent IBD and HF, it is evident that further research is necessary to fully understand the relationship between IBD and its cardiovascular outcomes.

### 3.1 Diagnosis and Treatment

IBD and CVD are commonly diagnosed through a comprehensive approach that involves the patient's medical history, physical examination, and various assessments that are selected based on the patient’s specific presentation. In the case of IBD, the diagnostic process includes the use of endoscopy (gold standard test), along with biopsy samples, obtained during the endoscopic procedure to further differentiate between CD and UC [49, 50]. On the other hand, the diagnosis of CVD typically entails a range of tests, such as electrocardiogram, echocardiogram, stress test, high-sensitivity troponin assays, biomarkers, cardiac catheterization, and coronary angiography [51]. Healthcare providers should be aware that pharmacotherapeutic agents commonly used to reduce inflammation and manage symptoms in IBD patients, such as corticosteroids, 5-aminosalicylic acid (5-ASA), thiopurines, and immunotherapies, like TNF-α or Janus kinase (JAK) inhibitors, may have implications for ASCVD [52, 53]. To illustrate, corticosteroids have been linked to an increased risk of heart failure, thromboembolism, and insulin resistance, while JAK inhibitors (including tofacitinib) further accentuate the risk for CVD by elevating lipid levels [54-56]. On the other hand, TNF-α inhibitors have been shown to decrease the risk of CVD by decreasing aortic stiffness and pro-coagulant levels, such as CRP and fibrinogen [57]. A study performed by Rungoe *et al.* suggests that treatment with 5-ASA drugs may reduce the overall cardiovascular risk and IHD in IBD patients compared to non-users; however, high doses of salicylates have been associated with complications, such as pericarditis and aortic stiffness [14, 58, 59]. To optimize treatment for IBD patients, healthcare providers should thoroughly assess for and be aware of potential CVD risks, and consider using appropriate diagnostics and specialized cardiac tests, and evaluating the potential cardiovascular risks and benefits of treatment. By comprehending and evaluating CVD risks in IBD patients, healthcare providers can personalize treatment plans to meet individual needs [55].

### 3.2 Future Directions

Multiple treatments are currently being investigated, which have shown some promise to improve the prognosis of IBD and its EIMs (including the development of CVD). Fecal microbiota transplant, in particular, has shown a higher likelihood of remission at 8 weeks in patients with mild to moderate UC endoscopic improvement and is associated with distinct microbial changes related to the outcome. That being said, further research is needed to assess longer-term maintenance of remission and safety and define optimum treatment intensity. Moving forward, it may also be helpful to develop and identify more biomarkers that can predict cardiovascular risk, and further investigation should be conducted to tailor treatment on an individual basis due to the risk of cardiovascular complications associated with some treatments of IBD.

## CONCLUSION

CD and UC are IBDs that can have many EIMs, including developing CVDs, such as ASCVD, pericarditis, myocarditis, pulmonary embolisms, stroke, arrhythmias, heart failure, and valvular abnormalities. These cardiovascular EIMs develop due to the various pathophysiological changes that occur in IBD, such as alterations of the gut microbiota, increased inflammatory cytokines and development of endotoxemia, changes in lipid metabolism, structural changes to the arterial wall, and having a hypercoagulable state. The primary treatment options for IBD include corticosteroids, 5-ASA and its derivatives, thiopurines, and immunotherapies, such as anti-TNF-α agents and JAK inhibitors. While these therapies are known to reduce inflammation and alleviate IBD symptoms, some may cause further cardiovascular complications. Therefore, healthcare providers should be aware of the association between IBD and CVD and do a full diagnostic workup prior to providing any interventions. Once the diagnostic workup is completed, a multidisciplinary approach that addresses gut inflammation while preventing and/or treating CVD should be taken to optimize treatment and reduce the morbidity and mortality of IBD-associated CVD. Additionally, lifestyle modifications, such as smoking cessation, regular physical activity, and dietary changes, may help decrease the risk of developing CVD in IBD patients. Given the increasing prevalence of IBD globally, further investigations should be performed to evaluate the association between IBD and CVD in order to provide better quality care for patients.

## LIST OF ABBREVIATIONS

5-ASA: 5-aminosalicylic Acid
ASCVD: Atherosclerotic Cardiovascular Disease
CD: Crohn's Disease
cfPWV: Carotid-femoral Pulse Wave Velocity
CIMT: Carotid Intima-media Thickness
CRP: C-reactive Protein
CVD: Cardiovascular Disease
EIM: Extraintestinal Manifestation
FMD: Flow-mediated Dilatation
IBD: Inflammatory Bowel Disease
IHD: Ischemic Heart Disease
IL: Interleukin
JAK: Janus Kinase
RR: Relative Risk
TNF-α: Tumor Necrosis Factor Alpha
UC: Ulcerative Colitis
